# Ecophysiological impacts of Esca, a devastating grapevine trunk disease, on *Vitis vinifera* L.

**DOI:** 10.1371/journal.pone.0222586

**Published:** 2019-09-19

**Authors:** Loris Ouadi, Emilie Bruez, Sylvie Bastien, Jessica Vallance, Pascal Lecomte, Jean-Christophe Domec, Patrice Rey

**Affiliations:** 1 INRA, ISVV, UMR1065 Santé et Agroécologie du Vignoble (SAVE), Villenave d'Ornon, France; 2 Bordeaux Sciences Agro, INRA UMR1391 Interactions Sol Plante Atmosphère (ISPA), Villenave d'Ornon, France; 3 Université de Bordeaux, ISVV, UR Œnologie, Villenave d’Ornon, France; 4 Université de Bordeaux, ISVV, UMR1065 Santé et Agroécologie du Vignoble (SAVE), Bordeaux Sciences Agro, Villenave d'Ornon, France; Universidade do Minho, PORTUGAL

## Abstract

Esca is a Grapevine Trunk Disease (GTD) caused by a broad range of taxonomically unrelated fungal pathogens. These attack grapevine wood tissues inducing necroses even in the conductive vascular tissues, thus affecting the vine physiology and potentially leading to plant death. However, the influence of Esca on leaf and whole-plant water transport disruption remains poorly understood. In this paper, a detailed analysis of xylem-related physiological parameters in grapevines that expressed Esca-foliar symptoms was carried out. The experiments were conducted in a vineyard in the Bordeaux region (France) on cv. Cabernet-Sauvignon (*Vitis vinifera* L.) grapevines, which were monitored for Esca-foliar symptoms over a two-year period. Heat dissipation sap-flow sensors were installed during the summer on grapevines having expressed or not Esca-foliar symptoms. Leaf water potential, stomatal conductance and leaf transpiration were also measured. Physiological monitoring showed that sap flow density and whole-plant transpiration of Esca-infected grapevines decreased significantly a week before the first foliar symptoms appeared. When atmospheric water demand (Vapour Pressure Deficit, VPD) was the highest, both parameters tended to be about twice as low in symptomatic grapevines as in asymptomatic ones. Sap flow density data at the maximum transpiration-time, was systematically 29–30% lower in Esca-infected grapevines compared to control plants before or after the appearance of Esca-foliar symptoms. This trend was observed whatever the temperatures and VPD values measured. In Esca-diseased plants, larger amounts of necrotic wood, mainly white rot, were found in the trunk and cordon of symptomatic grapevines compared to healthy ones, suggesting necroses have an influence in reducing the whole-plant hydraulic capacity. This study reveals that the use of physiological monitoring methods, together with the visual monitoring of foliar symptoms, could prove useful in providing accurate measurements of Esca disease severity.

## Introduction

Grapevine Trunk Diseases (GTDs) such as Esca, Eutypa and Botryosphaeria diebacks are among the most vineyard-destructive diseases, with their continuous spread threatening the sustainability of the vineyard worldwide. Over the last two decades, GTDs have become a major issue for the wine industry. The cost of replacing dead grapevines worldwide had been estimated at over 1.5 billion dollars per year [1], but that seems considerably underestimated when more local data are taken into account. In France, for instance, GTDs affect approximately 13% of the vineyards [2], with the French Wine Institute (IFV) estimating annual wine production losses at 1 billion euros. Extensive research has been carried out in recent years, with investigations focusing on epidemiology, aetiology, microbial ecology, host-pathogen relationships, and biocontrol [3–6]. So far, no effective alternative control methods are available for winegrowers [7], and no grapevine cultivars are known to be completely resistant to trunk diseases [8–11].

As regards Esca, the lack of effective control treatments has triggered great apprehension in the viticulture sector. Previously, sodium arsenite was the only pesticide registered in Europe to control Esca but it was banned in the early 2000s, because of its toxicity for both winegrowers and the environment [12,13,14]. However, the exponential growth of Esca symptoms has also been observed in countries that never used sodium arsenite (Germany: [15]; Switzerland: [16]). According to Fussler et al. [17], although sodium arsenite reduced the severity of Esca foliar symptoms, it had no incidence on plant mortality. Other factors, such as environmental ones or climate change, could be involved, but their role in the spread of the disease has not yet been determined.

Esca is a vascular wilt disease that attacks the perennial organs of grapevines, producing extensive wood necrosis in the trunk and cordon through the slow and systemic development of pathogenic fungi such as *Phaeomoniella chlamydospora*, *Phaeoacremonium minimum*, *Fomitiporia mediterranea* and *Botryosphaeriaceae* species [18–22]. Esca is a complex disease, involving various biotic factors (pathogenic fungi), and abiotic factors possibly associated with cultural practices [6,23,24]. Fungal colonization of the wood can reach a critical point when the functional tissues are severely damaged, thus possibly interfering with the vine physiology, leading to Esca-foliar symptom expression and/or ultimately to plant death. Parameters such as type of cultivar, pruning methods, climate are frequently listed as factors affecting the development of Esca [23,24]. Two main forms of the disease have been described: (i) a chronic (or slow) form, involving characteristic “tiger-stripe” discolouration of the leaves and (ii) an acute (or apoplectic) form, characterised by a sudden drying of the leaves during the hot season, rapidly leading to the death of the vine. In recent years, Lecomte et al. [6] introduced a classification based on a graduated scale of severity, going from leaves showing some discoloration to total vine wilting. However, as the manifestation of the foliar symptoms may fluctuate from year to year, it is difficult to estimate the real incidence of the disease based only on the annual expression of symptoms on the leaves.

It has been reported that the volume of necrotic wood determines the probability of leaf symptoms occurrence [25] and, the volume of white rot within the grapevine wood increases the risk of developing apoplectic forms [26]. Altogether, these results [25,26] indicate that limiting or reducing the development of wood necrosis during the life of a vine is a critical element for an effective control of the disease. Specifically, Esca pathogens involved in the development of wood necrosis show varying ability to utilize nutrients made available in injured tissues [6]. Higher nutrient availability in xylem sap could lead to increased fungal growth over time [27], and potentially affect sap transport in infected vines.

Although grapevine sap flow is extremely sensitive to changes in sapwood functionality as well as to sudden changes in xylem pressure [28], few studies have been carried out on the dysfunction of the vascular system in Esca-infected vines and its consequences on the whole-plant physiology. As shown with pathogens inoculations on trees, obstruction of sap-conducting vessels can completely block the sap flow and cause stomatal closure [29]. In grapevines, a similar hydraulic blockage, consequent to the formation of necrosis in the sapwood, could disrupt stomatal conductance, reduce photosynthesis and CO_2_ assimilation, restrain the production of sugars and other secondary compounds involved in plant resistance mechanisms against Esca-related pathogens.

In addition to whole-plant water use and leaf-level water status, in the present experiment, we also assessed the changes in the intrinsic Water Used Efficiency (iWUE) in Esca-diseased vs control grapevines by measuring the stomatal activity and the carbon concentration. Additionally, measurements of the combined variation of δ^13^C and δ^18^O allowed us to specify whether the modifications of iWUE, following changes in environmental conditions, had a stomatal or a biochemical (photosynthesis driven) origin [30,31].

As regards δ^13^C, in C3 plants such as grapevines, carbon and oxygen isotope ratios can be valuable indicators of grapevine water stress [32]. When water becomes a limiting factor, the stomata close during part of the day, thus slowing down CO_2_ exchanges between the leaves and the atmosphere, and limiting ^13^C isotopic discrimination [33,34]. Under these conditions, the ^13^C/^12^C ratio (δ^13^C) in the primary products of photosynthesis approaches the one found in atmospheric CO_2_ and varies according to the stress experienced by the plant [35–37]. Concerning δ^18^O, ^18^O enrichment in leaves can be induced by an increasing evaporative demand, and suggests that a corresponding increase in δ^13^C is indicative of a drought-induced stomatal closure [38]. The ^18^O/^16^O ratio (δ^18^O) of leaf cellulose is largely determined by the integrated leaf-to-air vapour pressure gradient during photosynthetic gas exchange [39], and can be used to investigate changes in iWUE.

The objective of this study was to provide relevant information on the effect of Esca on grapevine ecophysiology, mainly on whole-plant water transport, leaf-level water status and gas exchanges. In the present paper, a detailed analysis of xylem-related physiological parameters in Cabernet-Sauvignon grapevines from the Bordeaux region that expressed, or not, Esca-foliar symptoms, was carried out. By monitoring sapwood, stomatal conductance and leaf transpiration rate, between asymptomatic and Esca-infected vines, we aimed at identifying and quantifying the physiological dysfunctions caused by the wood necroses on the circulatory activity of the whole plant. Additionally, we also used stable carbon isotope ratios as an index of iWUE to show how differences in physiological performance are related to the Esca disease.

## Materials and methods

### Plant material

Bordeaux Sciences Agro, the owner of the Chateau Luchey-Halde vineyard (Mérignac, Bordeaux region, France), gave permission to conduct the study. No specific permissions were required for these research activities since Bordeaux Sciences Agro promotes this type of experimental project. The field studies did not involve endangered or protected species.

In the Chateau Luchey-Halde vineyard, the study was conducted on 16-year-old Cabernet Sauvignon grapevines (*Vitis vinifera* L.), grafted on 10114 MG rootstock and planted on a sandy clay soil. Eight grapevine plants were selected, among which four expressed Esca-foliar symptoms in 2015 and four did not. The four vines that had continuously expressed Esca-foliar symptoms for at least two years in a row (2015 and 2016) were considered as Esca-infected. Similarly, the four vines that remained asymptomatic during this two-year period were considered as healthy.

Phenological observations were performed weekly, based on the international BBCH scale (Biologische Bundesantalt, Bundessortenamt und Chemische Industrie) [40]. From 22^nd^ June 2016 at the phenological stage 75 (fruit about half final size) on the BBCH scale to 27^th^ July 2016 at stage 81 (beginning of ripening), three leaf-level physiological parameters were measured (stomatal conductance, leaf transpiration rates, and leaf water status that included stem and midday leaf water potentials). During this monitoring period, all experimentations were conducted on asymptomatic leaves. The first Esca-foliar symptoms were observed on 13^th^ July 2016 at stage 76 (development of fruit). Then, the plants were uprooted and for each sampled vine, cordons and trunk were cut longitudinally to visualize and evaluate the necrotic and non-necrotic wood tissues in function of their length and localization. On each grapevine, two fully developed leaves were gathered from the medial part of each cordon then stored at -20°C prior to elemental and isotopic analyses. Healthy sapwood samples were also collected in the trunk and cordon of each vine and stored at -20°C prior to elemental and isotopic analyses. Whole-plant water transport (stem sap flow) was monitored from 5^th^ May 2016 at stage 19 on the BBCH scale (first leaves fully expanded) to 28^th^ July 2016 (stage 81) and thus encompassed the leaf-level physiological measurements.

### Leaf gas exchange, water potentials and hydraulic conductance

Measurements of stomatal conductance (g_s_) and leaf transpiration (E_leaf_) were carried out with a Li-Cor 1600 steady state porometer (Li-Cor Corp., Lincoln, NE, USA) on each of the 8 sampled grapevines. Only sun-exposed and recently fully expanded leaves were measured, with a minimum of two replicates per cordon. The measurements were conducted every two days between 10:00 a.m. and 12:00 a.m., when both parameters reached their maximum values. As soon as Esca-foliar symptoms were noticed, the measurements were performed every day. However, because of the high frequency of phytosanitary treatments that occurred in the vineyard, which is common for this time of year in the Bordeaux wine region, a total of 13 days of measurements were possibly achieved.

For each grapevine, leaf water potentials were measured in the vineyard, on two fully-grown leaves sampled from each cordon [41]. The petiole was cut at its tip by a scalpel to preserve the tissues then introduced into the lid opening of a pressure chamber (SAM Précis 2000, Gradignan, France). Three types of water potentials were measured: 1) predawn water potential (ψ _pd_) between 4:00 a.m. and 5:00 a.m., when the stomata are closed, and transpiration is negligible; 2) midday stem water potential between 10:00 a.m. and 11:00 a.m. on leaves that had been covered by an opaque, waterproof bag of aluminium at least one hour before the sampling [42], and 3) midday leaf water potential measured between 12:00 a.m. and 1:00 p.m. on sun-exposed leaves. For each day of measurement, gas exchange and water potentials were recorded concurrently to estimate whole-vine hydraulic conductance (K_vine_) from the relationship between the rates of single-leaf E_leaf_ and soil–leaf water potential difference [43]. The soil water potential (ψ _soil_) was considered to be very close to ψ _pd_ in the leaves [44], and thus K_vine_ was taken as:

$$Kvine=\frac{Eleaf}{\left(\psi pd-\psi leaf\right)}$$ (1)

Similarly, leaf hydraulic conductance (K_leaf_) was taken as:

$$Kleaf=\frac{Eleaf}{\psi stem-\psi leaf}$$ (2)

### Sap flow and evapotranspiration measurements

The first day of sap flow measurement started May 5, 2016 (stage 19 on the BBCH scale), we measured sap flux density per unit of conducting xylem area (J_s_, gH_2_O.m^-2^.s^-1^) on the eight selected grapevines. Stem sap flux measurements were made at 0.4 m above the ground using 10 mm heat dissipation probes [45], modified after the original 20 mm Granier’s design [46]. Preliminary results from a nearby plot showed that there was no significant difference in azimuthal J_s_ within grapevines (p = 0.42, Student’s paired t-test), probably as a result of sapwood homogeneity, so we inserted all probes on the north side of the vines. To account for natural axial gradients in temperatures between the heated and the reference probe due to soil heat exchange and therefore to reduce measurement noise [47], two additional and non-heated probes were inserted 4 cm to the right side of the primary probes [48]. In addition, stems were wrapped with reflective insulation using an aluminium sheath to minimize direct solar irradiance around the probes.

The sap flow density was estimated by continuously measuring the variations in temperature differences (ΔT) between a heating sensor operating at constant power and an identical non-heating sensor inserted upstream. The temperature of the heating sensor, maximum when the value of the sap flow is zero, decreases as the sap velocity grows, an increasing amount of heat being evacuated by convection. The signals from the sap flow probes were scanned every 30 seconds, and thirty-minute averages of temperature difference data were computed and stored in data loggers (CR1000; Campbell Scientific, USA) equipped with a 32-channel multiplexer (AM416; Campbell Scientific). The sensor signal was converted to J_s_, according to [49] and accounted for the effects of nonzero night-time fluxes on the signal baseline by using nights with low vapour pressure deficit (VPD) to estimate maximum temperature differences on nights with nocturnal transpiration [50].

Using sapwood area of each grapevine and stand grapevine density, J_s_ was scaled and converted to a plot scale average transpiration per unit ground area and per day (*TP*, in mm.d^-1^ corresponding to kg.m^-2^.d^-1^). From one day to the next, variations in the water content of the aerial part of grapevines are generally neglected as water storage compartment are refilled at night [42] and the daily TP was assimilated to the total sap flow cumulated over 24 hours as [51]:

$$TP=\sum_{t=1}^{24h}(J_{s}.3600)(S).10^{-3}$$ (3)

where S is the total grapevine conductive surface area per soil area. First, the total sapwood area of the grapevines equipped with sap flow probes was estimated from the relationship between sapwood area and diameter. Then this relationship was applied to the 79 grapevines surrounding the plants equipped with sapflow sensors to calculate whole plot sapwood area and by proportionality estimate whole plot *TP* using vine planting density.

### Necroses image-analyses

**Fig 1:**
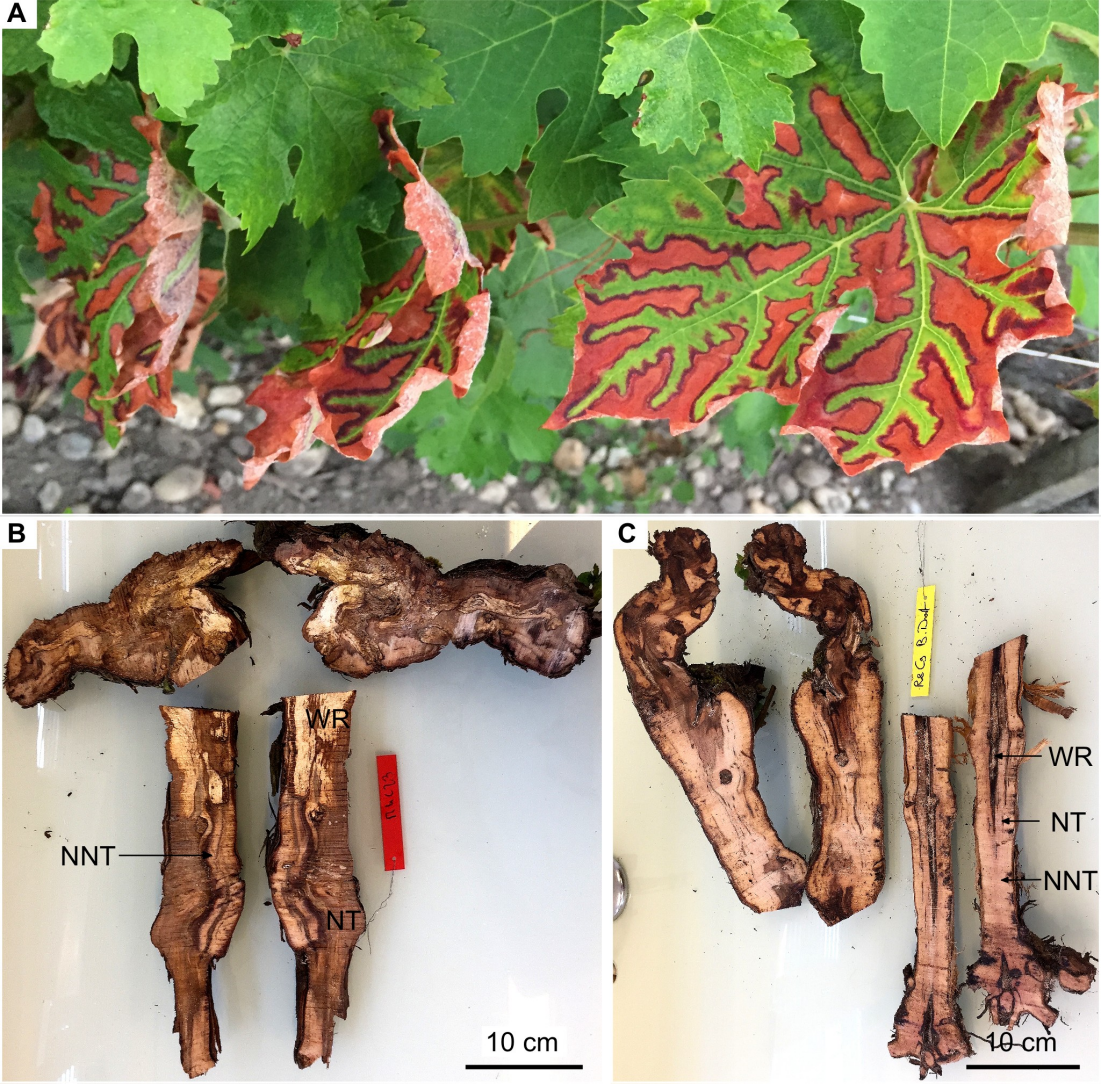
Symptoms expressions in Esca-infected Cabernet Sauvignon grapevines. (A) Grapevine showing tiger-stripe leaf symptoms associated with the chronic form of Esca. (B) Longitudinal section of an Esca-infected grapevine. Note the large area occupied by necrotic tissue (NT) and white rot (WR). (C) Longitudinal section of an asymptomatic grapevine in which most of the tissue is non-necrotic (NNT).

Once the physiological monitoring period was achieved, grapevines equipped with sapflow sensors were uprooted and cut longitudinally. One half of each trunk was then photographed for quantification of necrotic wood, which was, in comparison to apparently healthy wood, darker brown in colour and varied in texture from hard (necrotic-tissue) to soft and spongy (white-rot) (Fig 1). Necroses were evaluated according to the percentage of trunk area infected following [52]. The severity of each necrotic area was assessed from the photographs using the image-analysis software ImageJ (NIH, USA; https://imagej.nih.gov/ij/)

### Leaf nitrogen concentration and stable isotope measurements

Leaf chemistry as well as stable carbon and oxygen isotopes were used to evaluate grapevine water use efficiency. Both leaves and wood samples were collected for nitrogen (N) concentration (in grams of N per dry gram of sample, and converted to %N), and for isotopic measurements of δ^13^C. For isotopic measurements of δ^18^O, only leaf tissues were used. As some grapevines only expressed Esca-foliar symptoms on one of their cordons, leaf samples were differentiated according to the cordon they were collected from. Specifically, two leaves per cordon were collected from each sampled vine on 27^th^ July 2016 (stage 81 on the BBCH scale), before being ground in liquid nitrogen using a pestle and mortar then stored at– 20°C before downstream analyses.

Similarly, following the uprooting of grapevine plants that occurred just after the leaf harvest, non-necrotic woody tissues were collected on the trunk and cordon of each sampled grapevine before being ground in liquid nitrogen with a one-ball mill of Dangoumau type and kept at– 20°C prior to molecular analyses. After they were placed in the oven at 28°C overnight, 3 mg of each sample were weighed on an analytical balance and encased in tin foil capsules for both the determination of carbon and nitrogen concentrations and δ^13^C analysis. A second set of leaf samples intended for δ^18^O analysis was encased in silver capsules. The capsules were placed in a thermally sealed microplate and sent to the INRA-Nancy (France) for analysis. The δ^13^C analyses were performed with an Elementar Analyser (Carlo Erba, Milano) coupled to a Finnigan continuous flow isotope ratio mass spectrometer (Delta S, Finnigan MAT, Bremen, Germany) and the δ^18^O analyses were performed with a Pyrocube (Elementar, Hanau) coupled to an Isoprime (Manchester) at the stable isotope facility at Functional Ecology PTEF (OC 081) at INRA Nancy, France. The carbon isotopic composition was expressed according to the conventional δ (‰) notation:

$$\delta 13C=\left[\frac{RS-RPDB}{RPDB}\right]*1000$$ (4)

where R_S_ and R_PDB_ are the molar abundance ratios of the carbon isotopes ^13^C/^12^C in the sample and in the international standard for carbon called PDB (Pee Dee Belemnite) respectively [35]. The results were expressed in terms of the isotopic composition difference with the PDB (δ^13^C PDB). According to [53], δ^13^C PDB values of CO_2_ are around -8‰ in the atmosphere and in plants vary from -9 to -34‰.

### Statistical analysis

Statistical analyses were performed in R software [54]. In order to determine whether or not our variables of interest were different according to the health status of the grapevines, analyses of variance (ANOVA) were performed for each physiological parameter. The normality of the residuals of the variables was tested using the Schapiro-Wilk test. When the validation tests were not significant, a non-parametric test was used (Kruskal-Wallis). The tests were taken as significant when p<0.05.

## Results

### Esca-foliar symptoms and necrotic wood ratio

Before being uprooted, field notations were performed on the four asymptomatic (A1 to A4) and symptomatic (S1 to S4) grapevines selected for the experiment, to assess the presence of Esca-foliar symptoms (Table 1). Over the two-year monitoring period, changes in the expression of Esca disease occurred on S2. In 2015, only the left cordon of S2 showed Esca-foliar symptoms, while both cordons presented symptomatic leaves in 2016. The left cordon of S4 suffered the apoplectic form of Esca in 2015 and is referred as dead (Table 1). The right cordon of A3 was torn out during the surveillance period and is referred as missing (Table 1).

**Table 1:** Notation table presenting the status of the 16-year-old Cabernet-Sauvignon cultivar (*Vitis vinifera* L.) sampled in 2015 and 2016. In the table, (-) stands for asymptomatic cordons and (+) stands for cordons that expressed Esca-foliar symptoms.

Vine		Esca-foliar symptoms in 2015		Esca-foliar symptoms in 2016	
		Left cordon	Right cordon	Left cordon	Right cordon
Asymptomatic	A1	-	-	-	-
	A2	-	-	-	-
	A3	-	-	-	Missing
	A4	-	-	-	-
Symptomatic	S1	+	-	+	-
	S2	+	-	+	+
	S3	+	+	+	+
	S4	Dead	+	Dead	+

**Fig 2:**
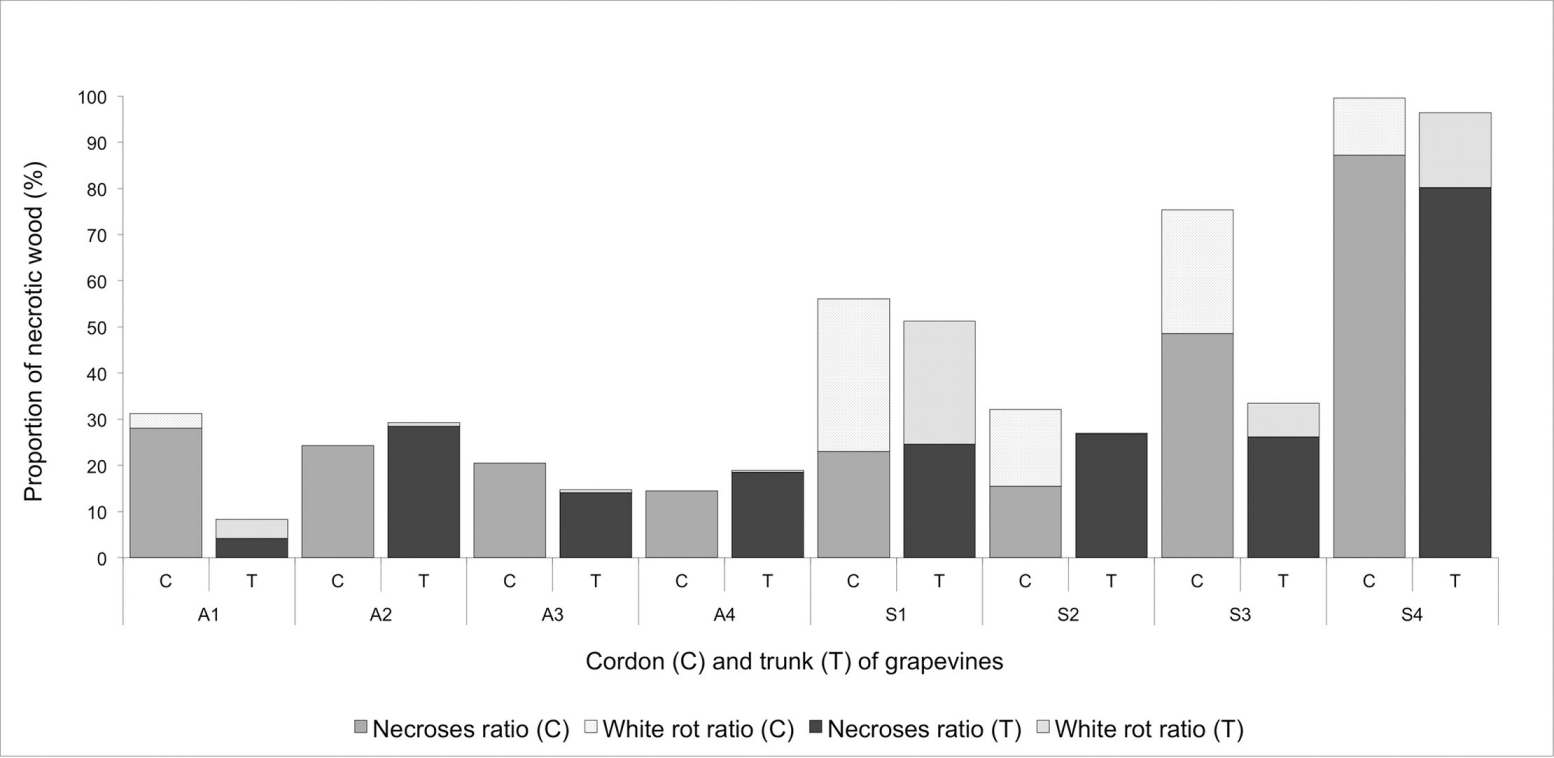
Percentage of necrosis and white rot in the trunk wood (T) and cordons (C) of asymptomatic (A) and symptomatic (S) grapevines.

When looking at the whole grapevine, trunk and cordon combined, larger amounts of necrotic-wood including white rot were found inside grapevines that expressed Esca-foliar symptoms (Fig 2). In symptomatic plants, white rot ratios consistently accounted for 15% to 50% of the total necrotic area regardless of the tissue considered. Even though no trace of white rot was found in the trunk of S2, when considering the whole plant, the share of white rot remained preponderant in comparison to the healthy grapevines. In asymptomatic grapevines, the necrotic-wood ratio remained low with less than 30% of the total wood surface being necrotic, and with a negligible proportion of white rot. The grapevine A1 that was healthy during the two-year survey had a total necrotic-wood ratio close to the other asymptomatic grapevines. Yet it was the only healthy grapevine that presented traces of white rot, accounting for less than 5% of the total wood surface in both the trunk and cordons.

### Sap flow density and grapevine water use

The data selected to analyse the variation of sap flow were acquired continuously between stage 19 on the BBCH scale (10 leaves unfolded, i.e. early May) and stage 81 (beginning of ripening, i.e. late July), mostly when no Esca-foliar symptoms had yet appeared in the field, since the first symptoms were observed at stage 76 (development of fruit). No mechanical leaf pruning occurred between those dates. Two different sets of environmental conditions characterized the data-recording phase. The first period ranged from stage 61 (beginning of flowering, i.e. late May) to stage 76 (fruit about half-size; i.e. early July), when temperatures did not exceed 27°C and the mean maximum daily Vapour-Pressure Deficit (VPD) remained below 1.2 kPa. The second period starting at the end of stage 76 was marked by higher temperatures and evaporative demand, where midday VPD values were twice as high.

**Fig 3:**
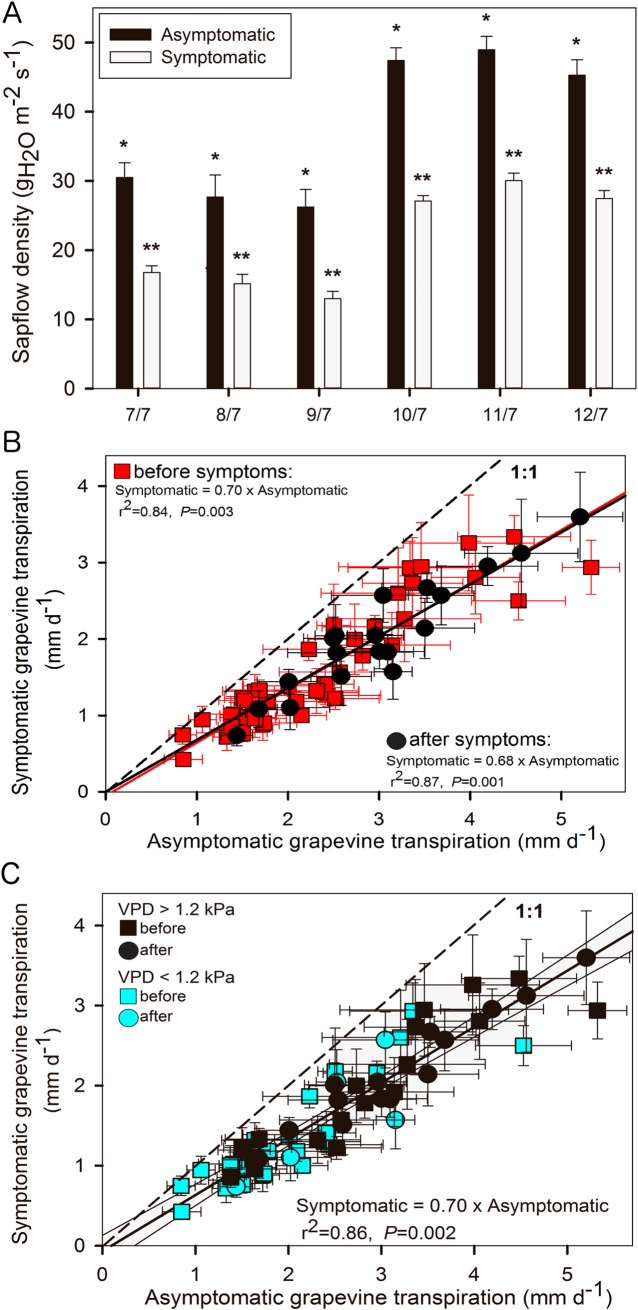
Comparison of daily sap flow densities and whole plant transpiration in asymptomatic and symptomatic Cabernet Sauvignon grapevines. (A) Mean maximum daily sap flow densities in asymptomatic and symptomatic grapevines during the week preceding the onset of Esca-foliar symptoms. Data represent transpiration values under non-limited light conditions, i.e. recorded between 10:00 a.m. and 4:00 p.m. Error bars represent SE and a different number of stars for a given date indicates a significant difference between asymptomatic and symptomatic grapevines at p<0.05. (B) Mean daily transpiration in asymptomatic and symptomatic grapevines. Data were separated to reflect days “before” (red squares) and “after” (black circles) the apparition of the foliar Esca symptoms. Error bars represent SE. (C) Mean daily transpiration in asymptomatic and symptomatic grapevines. Data were separated to reflect days with high (black symbols) and low (blue symbols) maximum vapour pressure deficit (VPD), and to represent days “before” (squares) and “after” (circles) the apparition of the foliar Esca symptoms. Error bars represent SE.

During stage 76 the diurnal time course of sap flux density reflected diurnal changes in ambient temperature and VPD. Note that no rainfall occurred over the 6 days preceding the onset of Esca-foliar symptoms. Sap flux density increased shortly after sunrise, reached a peak by 2:00 p.m. and decreased in the late afternoon (S1 Fig). No rainfall was recorded during this period (S2 Fig). The analysis of sap flow dynamics according to the health status of grapevines, one week before the onset of the first Esca-foliar symptoms, revealed two distinct kinetics. Although sap flow densities recorded in Esca-infected and control grapevines show a similar evolution, the average flow velocity measured around midday periods in grapevines that were about to develop Esca-foliar symptoms was significantly lower (p<0.05), compared to controls plants. The end of stage 76, characterized by a warmer, drier climate than the previous days, sap flow densities in both control and symptomatic grapevines were amplified (Fig 3A). However, sap flow densities and whole-plant transpiration of grapevines that developed Esca-foliar symptoms were consistently lower in comparison with the control plants (Fig 3). To better estimate the water balance in symptomatic and asymptomatic plants, the conductive tissue area of each grapevine was taken into account to calculate the daily transpiration (Fig 3B, Fig 3C). On average, the transpiration ranged from 2 to 5 mm per day. The daily transpiration recorded in symptomatic grapevines was about 30% lower than in healthy plants (p = 0.002). This downward trend remained the same before and after the apparition of the foliar Esca symptoms (Fig 3B). Additionally, the relationship between transpiration values in asymptomatic vs. symptomatic grapevines did not appear to be particularly modified when climatic conditions were characterized by higher VPD (Fig 3C).

### Leaf stomatal conductance, transpiration, and water status

**Fig 4:**
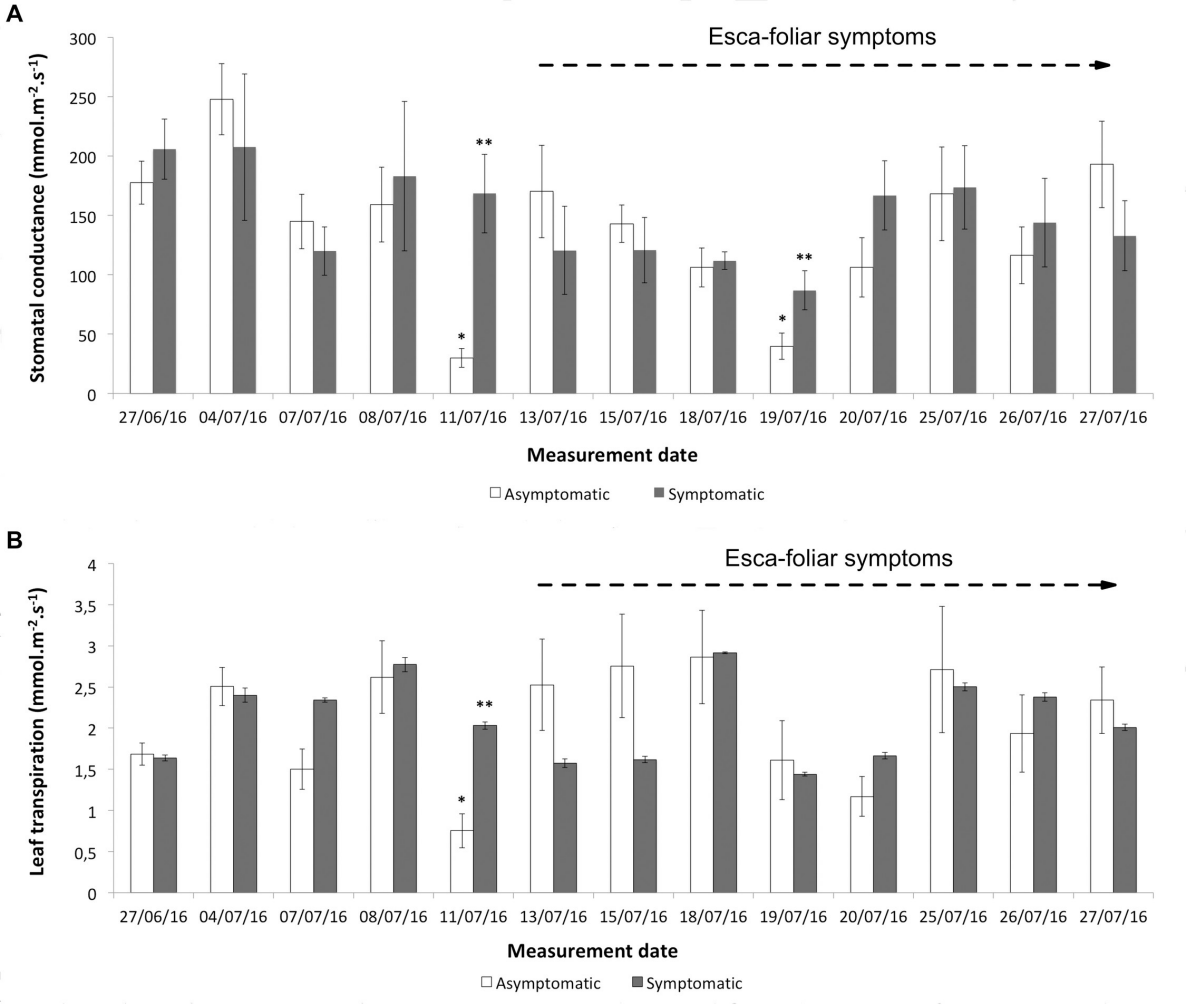
Evolution of stomatal conductance and leaf transpiration in asymptomatic and symptomatic Cabernet Sauvignon grapevines. (A) Average stomatal conductance in asymptomatic and symptomatic grapevines. (B) Average foliar transpiration in asymptomatic and symptomatic grapevines. The first Esca-foliar symptoms were observed on July 13^th^ (stage 76 on the BBCH scale). Error bars represent SE and a different number of stars for a given date indicates a significant difference between asymptomatic and symptomatic grapevines at p<0.05.

From stage 75 on the BBCH scale to stage 76, when grapevines had not yet expressed any leaf symptoms, the average g_s_ was significantly higher (p = 0.04) in Esca-infected grapevines than in control plants (169.3 mmol.m^-2^.s^-1^ vs 28.2 mmol.m^-2^.s^-1^, respectively) only two days before the first symptoms appeared (Fig 4A). This difference was also recorded at stage 77, one week after the onset of Esca-foliar symptoms (p = 0.021; Fig 4A). Consequently, E_leaf_ measured on that day on symptomatic leaves was also significantly higher (2.05 mmol.m^-2^.s^-1^) than those from the asymptomatic (0.78 mmol.m^-2^.s^-1^) ones (p = 0.06; Fig 4B). After stage 81 (beginning of ripening), affected leaves started to significantly dry and curl, preventing accurate measurement of leaf transpiration (E_leaf_) and stomatal conductance (g_s_).

**Fig 5:**
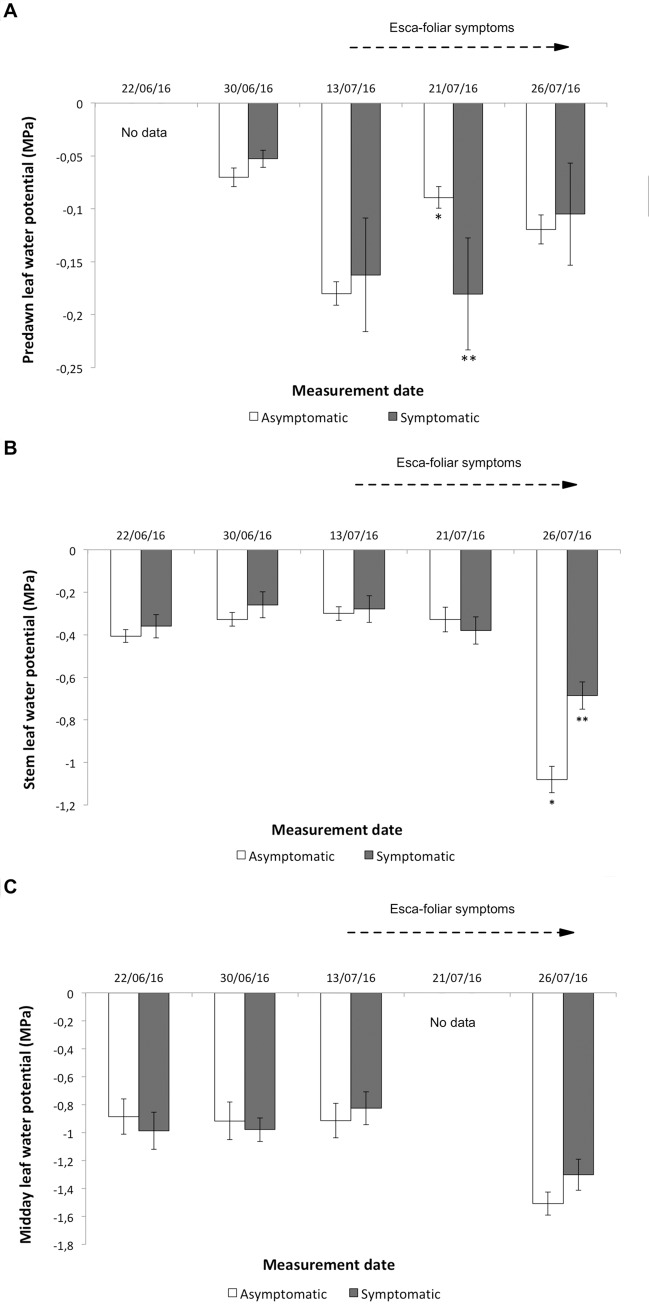
Evolution of water potentials in asymptomatic and symptomatic Cabernet Sauvignon grapevines. (A) Predawn leaf water potentials, (B) stem leaf water potentials, and (C) midday leaf water potentials in asymptomatic and symptomatic grapevines. The first Esca-foliar symptoms were observed on July 13^th^ (stage 76 on the BBCH scale). No data were recorded for predawn water potential on June 22nd (A) and for leaf water potential on July 21st (C) due to bad weather conditions. Error bars represent SE. The different number of stars indicates a significant difference between asymptomatic and symptomatic grapevines at p<0.05.

No conclusive and consistent differences in leaf water potentials between asymptomatic and symptomatic grapevines were recorded over the physiological monitoring period. For example, predawn water potential measured at the phenological stage 77 on the BBCH scale (bunch closure), was twice as low in symptomatic grapevines than in control plants, but then five days later those values were similar (Fig 5A). At the beginning of ripening (stage 81), stem leaf water potentials showed lower values in asymptomatic grapevines in comparison with healthy plants recorded (Fig 5B). No significant differences were obtained with midday leaf water potential (Fig 5C). Even though grapevines did not suffer from water stress as seen in the similar values of whole-vine hydraulic conductance (K_vine_), significant differences in leaf water status and leaf hydraulic conductance (K_leaf_) were recorded between symptomatic and asymptomatic plants (Table 2). As a consequence the leaf resistance of symptomatic vines increased to more than 70% of the total root-to leaf hydraulic resistance.

**Table 2:** Impacts of the grapevine trunk disease Esca on hydraulic conductance of whole-vine (K_vine_) and leaf (K_leaf_) of Cabernet-Sauvignon cultivar (*Vitis vinifera* L.). Values shown are means from three different sampling dates where gas exchanges and water potentials were measured concurrently (from the end of flowering to berry ripening). Different letters within a row indicate a significant effect at p<0.05.

	Asymptomatic	Symptomatic
K_vine_ (mmol m^-2^ MPa^-1^ s^-1^)	3.0 ± 0.6 **a**	2.4 ± 0.3 **a**
K_leaf_ (mmol m^-2^ MPa^-1^ s^-1^)	5.0 ±0.3 **a**	3.4 ± 0.4 **b**
Resistance in leaves (%)	61 ± 15 **b**	72 ± 10 **a**

### Carbon, nitrogen concentrations and stable isotopes

**Fig 6:**
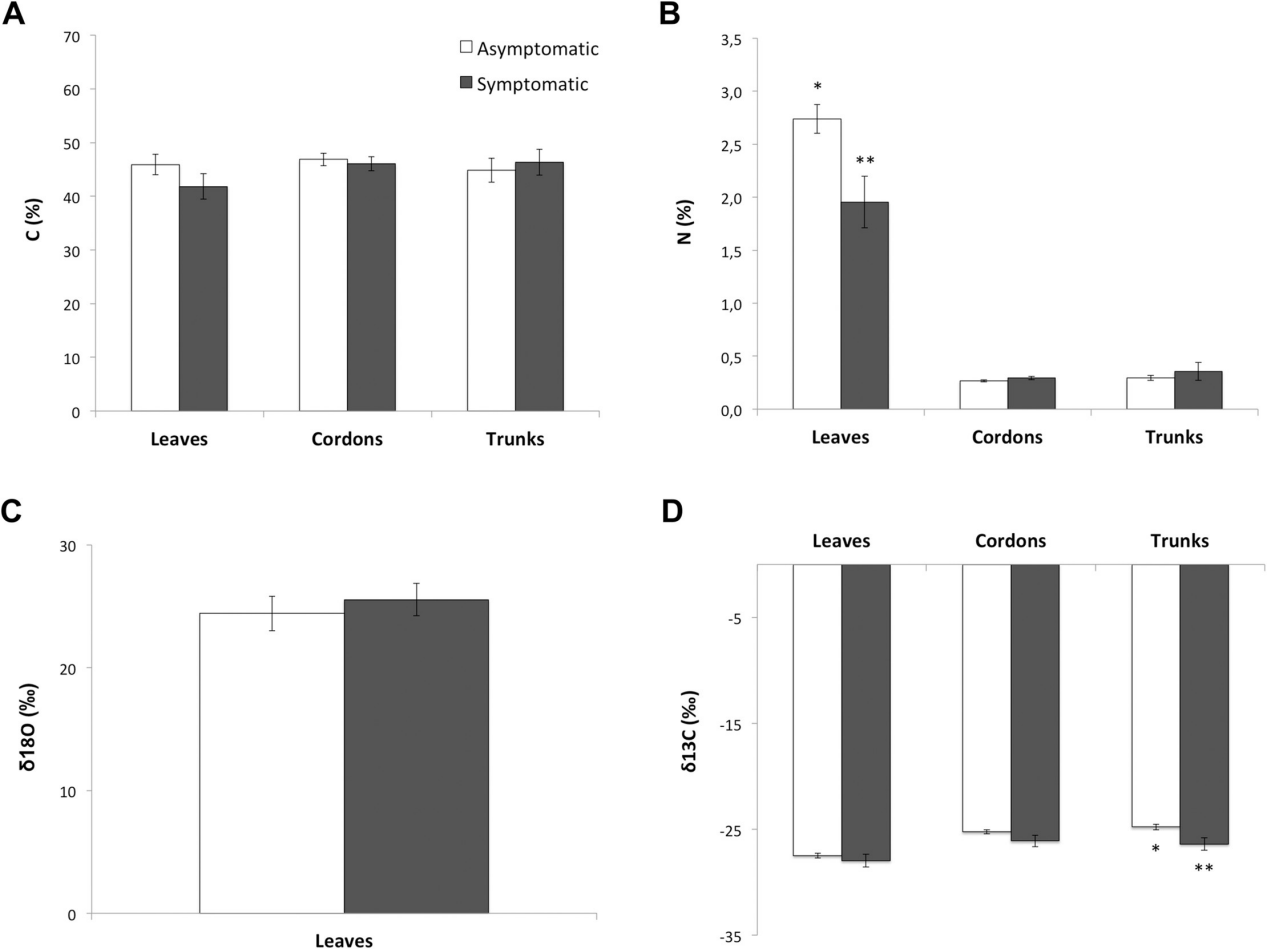
Elemental and isotopic analysis of leaf, cordon and trunk samples collected from symptomatic and asymptomatic grapevines. (A) Percentage of carbon and (B) percentage of nitrogen in leaves, cordons and trunks of symptomatic and asymptomatic grapevines. (C) Oxygen isotope discrimination (δ^18^O) in leaves and (D) carbon (δ^13^C) isotope discrimination in leaves, cordons and trunk of symptomatic and asymptomatic grapevines. For each type of measurement, non-necrotic samples were used. White Error bars represent SE and a different number of stars for a given tissue indicates a significant difference between asymptomatic (white columns) and symptomatic (black columns) grapevines at p<0.05.

There was no difference in carbon concentration (%C) in leaves, cordons and trunks between healthy and Esca-infected leaves (Fig 6A). However, nitrogen concentration (%N) of leaves that developed Esca-foliar symptoms in 2015 and 2016 was significantly lower (p = 0.03) in symptomatic (N = 1.96 ± 0.24%) than in control plants (N = 2.74 ± 0.14%) (Fig 6B). There was no difference in foliar oxygen isotope ratios δ^18^O between healthy and infected vines (Fig 6C). Isotopic fractionation resulted in some quantitative differences in δ^13^C between organs, with cordon and trunk signals being less negative, than leaves. Carbon isotope ratios δ^13^C of necrotic wood tissues sampled from the trunk of symptomatic grapevines (δ^13^C = -26.4 ± 0.58‰) was significantly more negative (p = 0.04) than in healthy grapevines (δ^13^C = -24.8 ± 0.26‰), indicative of a decrease in instantaneous water use efficiency (iWUE) in Esca infested vines (Fig 6D).

## Discussion

The image analyses that were performed on the longitudinal sections of grapevines revealed that the level of severity of internal necroses, mainly white rot, was higher in plants that had expressed Esca-foliar symptoms the two last years. As previously reported by Maler et al. [26], those results confirmed that the value of at least 10% of white rot in the cordons of grapevines is a strong descriptor for the chronic form of Esca.

The originality of this study lies in the adaptation of a physiological monitoring tool, mostly used on trees, to grapevine plants [55]. Thermal sapflow sensors adapted following Granier’s design [46,49] were installed on asymptomatic and Esca-infected grapevine and different dynamics in sap flow density were recorded. Regardless of the date of appearance of Esca-foliar symptoms, sap flow density data around the maximum transpiration-time, was systematically about 30% lower in Esca-infected grapevines compared to control plants. This trend was observed whatever the temperatures and VPD values measured. Regardless of the health status of grapevines, the higher levels of sap flow density were recorded when the evaporative demand was particularly strong.

Results in grapevine were consistent with those of the literature that indicate that sap flow sensors are able to detect changes in hydraulic function prior to the onset of obvious visual symptoms, e.g. in walnut trees [56]. Here, the continuous physiological monitoring of sap flow made it possible to demonstrate changes, i.e. decrease in the water transport within Esca-diseased grapevines. Based on the greater amount of wood necroses in these plants, it can be suggested that the vascular system is altered. As a consequence, the loss of grapevine capacity to transport water probably results from vessel occlusion due to the internal necrosis and the formation of tyloses and gels within vessels [57]. The number of functional vessels thus decreases and the circulatory activity of the whole plant is hindered.

The development of a non-destructive monitoring method to detect the early ecophysiological changes occurring in Esca-infected vines could improve the accuracy of the epidemiological surveillance network by adding key information on the plant functioning, beside the foliar symptoms that are currently monitored in GTD-epidemiological survey. A detailed quantification of xylem-related physiological parameters in the trunk and on the leaves of grapevines would also potentially provide accurate measurements of the disease severity.

Data recorded at the leaf level, i.e. stomatal conductance, leaf transpiration and water potential, leaf hydraulic conductance, were more difficult to analyse than those at the whole-plant level (from the sap flow sensors) due to the high variability of the measurements. Except for a few days, no clear significant differences were measured in grapevine expressing Esca-foliar symptoms and control plants, but other studies have observed differences. According to Petit et al. [58], Esca-symptomatic leaves are associated with both stomatal closure and alteration of the photosynthetic apparatus. However, the physiological mechanisms underlying the appearance of symptoms have not been yet identified. In this study, all measurements were performed on healthy leaves and no recurrent differences in g_s_ were recorded between Esca-infected grapevines and controls over the study period. During the season, the development of foliar symptoms in infected plants reduces their functional leaf area, as characterized by the tiger-stripe leaf symptoms (Fig 1). This loss of leaf active surface, associated with the decrease in sapflow densities recorded in symptomatic grapevines could explain why g_s_ remained nearly constant between healthy and infected grapevines. As shown by Pataky et al. [59], under identical conditions of temperature and humidity g_s_ within a plant is directly proportional to transpiration and inversely proportional to leaf area. Therefore, it is likely that the decrease in whole-vine sapflow and transpiration in infected vines (~30%, Fig 3) due to wood damage (Fig 2) was likely compensated by a proportional reduction in active foliage, thus maintaining a constant g_s_. (Fig 1)

In 2007, Edwards et al. [60] measured an increase of the leaf stomatal conductance in grapevine, which led directly to a water deficit (estimated by lower water potentials) in response to *P*. *chlamydospora* infections in 3-year-old potted plants that were maintained in greenhouse conditions. Our results can be explained by the environmental conditions in the vineyard that change from day to day, whereas they are much more stable and regulated in the greenhouse. Our results can also be regarded as the consequence of enlightenment, which is not high enough, to induce significant differences in the diseased and control plants, particularly before the onset of the foliar symptoms.

Predawn water potential measurements are assumed to be an indicator of available water in the soil [61]. The more negative predawn water potential of symptomatic grapevines just after the first Esca-foliar symptoms appearance indicated that infestation must have caused a form of resistance in the roots. This hypothesis is supported by the fact that all these grapevines, i.e. diseased and control plants, were growing on the same site. It has been shown that, if roots are affected at the soil–root interface, soil-root resistance will increase and the ability of plants to take up water from the soil will diminished [62], as seen with the sap flow data. For instance, during a drought event, diseased-grapevines could be further compromised because of this added resistance.

Here, this increase in resistance was supported by the higher percentage of resistance that was measured on the leaves of Esca-diseased grapevines. Leaves comprise the terminal portion of the liquid water transport pathway and their xylem is under greater tension than in stems. The decline in hydraulic conductance of leaves (K_leaf_) in Esca-foliar symptomatic grapevines was not the consequence of more native leaf water potentials, therefore some leaf structural adjustments explaining this increase in leaf hydraulic resistance occurred following leaf symptomatic expression. Although the hydraulic system of leaves represents less than 5% of the total hydraulic pathway [63,64], we showed that a substantial (50–80%) and variable part of the resistance to water flow was located in the leaves. The tight coordination that exists between K_leaf_ and stomatal conductance (g_s_) within and among species [65] implies that the large declines of K_leaf_ in Esca-diseased grapevines may be an inherent component of the stomatal regulatory system acting as a signal to reduce water flow.

To go further, measurements of stomatal conductance and carbon assimilation were used to estimate the instantaneous intrinsic Water Use Efficiency (iWUE). The combined variation of δ^13^C and δ^18^O allowed to specify whether the modifications in iWUE following changes in environmental conditions have a biochemical (photosynthesis driven) or a stomatal origin [30,31]. Unlike assessment of δ^13^C in leaves, stem δ^13^C measurements are less subject to time and daily climatic constraints, and reflect an integrative growing condition of the grapevines. The decrease in trunk δ^13^C for the Esca diseased-grapevines indicated that over the long-term, iWUE decreased in these plants [33,66]. As for δ^18^O, there were no significant correlations differences in δ^18^O between Esca-symptomatic and asymptomatic grapevines, suggesting that the variability in δ^13^C was not driven by differences in g_s_, but rather by reduced carbon assimilation, (i.e. a decrease in photosynthesis) [67]. This conclusion was supported by the lower nitrogen concentration, which means lower Rubisco activity, found in leaves that developed Esca-foliar symptoms than in control plants.

## Conclusion

This study shows that the use of a physiological monitoring method to record changes in sap flow of grapevine over time is of the utmost interest to provide accurate information on Esca-disease severity. To go further, we suggest that the use of sap flow sensors over several years on a same group of Esca-infected and healthy grapevines would create a significant database allowing an in-depth analysis of the physiological response in terms of water transport efficiency. This could be of particular interest when grapevine that is Esca-symptomatic in year n, become asymptomatic in year n+1, then asymptomatic or symptomatic in year n+2, etc. Sap flow measurements over time of these plants will allow to report if “symptomatic then asymptomatic ones” have the same level of hydraulic conductance than “always healthy plants” or if they are suffering irreversible alterations and are thus, more susceptible to die in the coming years. This knowledge would have a significant impact on the management of grapevines expressing foliar symptoms of Esca at the vineyard.

## Supporting information

S1 Fig Evolution of sap flow densities in asymptomatic and symptomatic grapevines under different climatic conditions. (A) Diurnal courses of sap flow densities in asymptomatic and symptomatic grapevines during the week preceding the onset of Esca-foliar symptoms. (B) Evolution of temperature and vapour-pressure deficit conditions during the week preceding the onset of Esca-foliar symptoms. Error bars in panel (A) represent SE.
(TIF)

S2 Fig Total weekly rainfall (mm) during the 2016 grapevine growing season (1^st^ March– 30^th^ September). (TIF)
