# Supplementary figures and images for: Ecophysiological impacts of Esca, a devastating grapevine trunk disease, on Vitis vinifera L
Source: PLoS One. 2019 Sep 19;14(9):e0222586. doi: 10.1371/journal.pone.0222586 (PMC6752872; doi:10.1371/journal.pone.0222586)

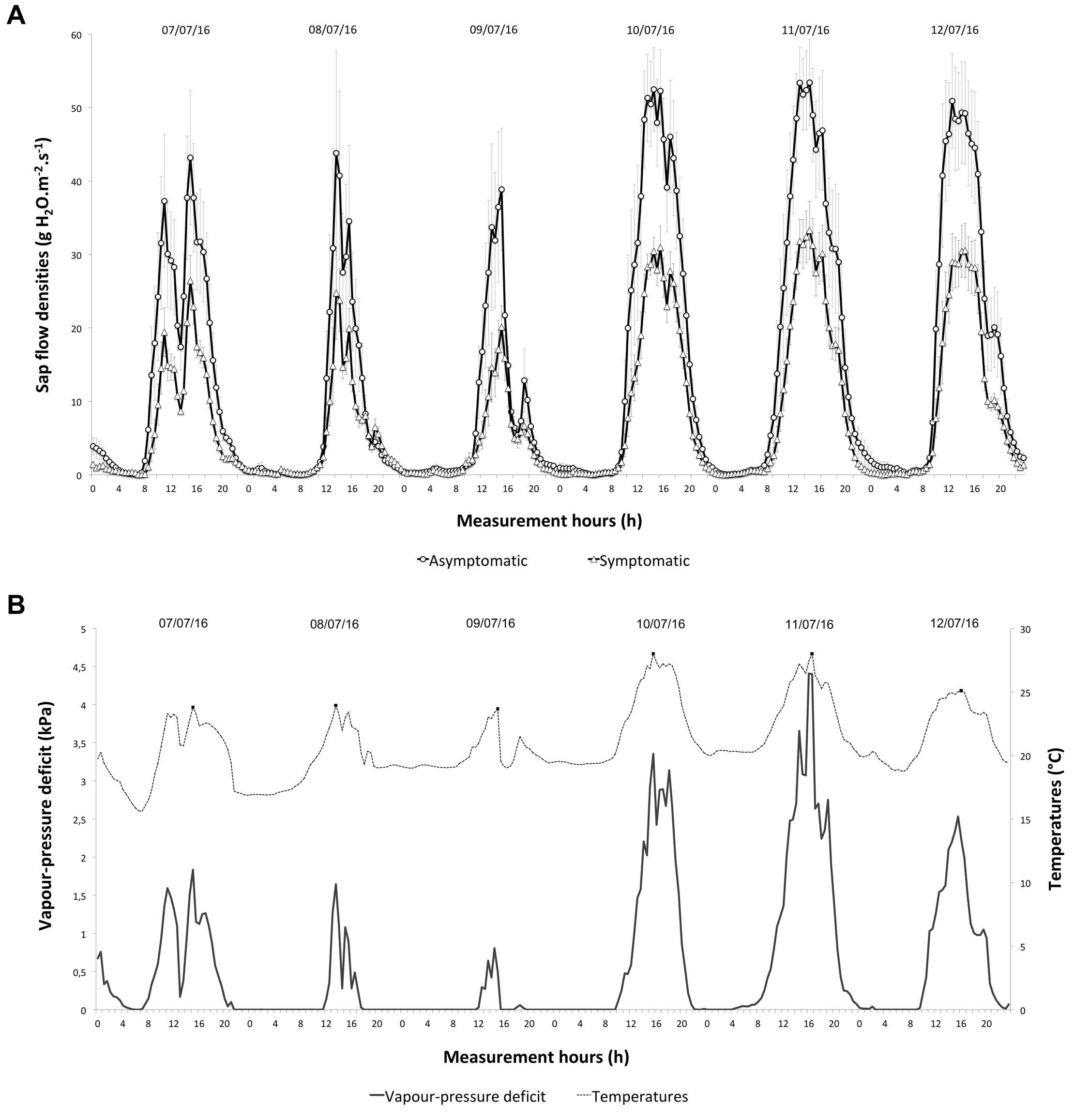

Supplement: S1 Fig — (A) Diurnal courses of sap flow densities in asymptomatic and symptomatic grapevines during the week preceding the onset of Esca-foliar symptoms. (B) Evolution of temperature and vapour-pressure deficit conditions during the week preceding the onset of Esca-foliar symptoms. Error bars in panel (A) represent SE. (TIF) [file pone.0222586.s001.tif]

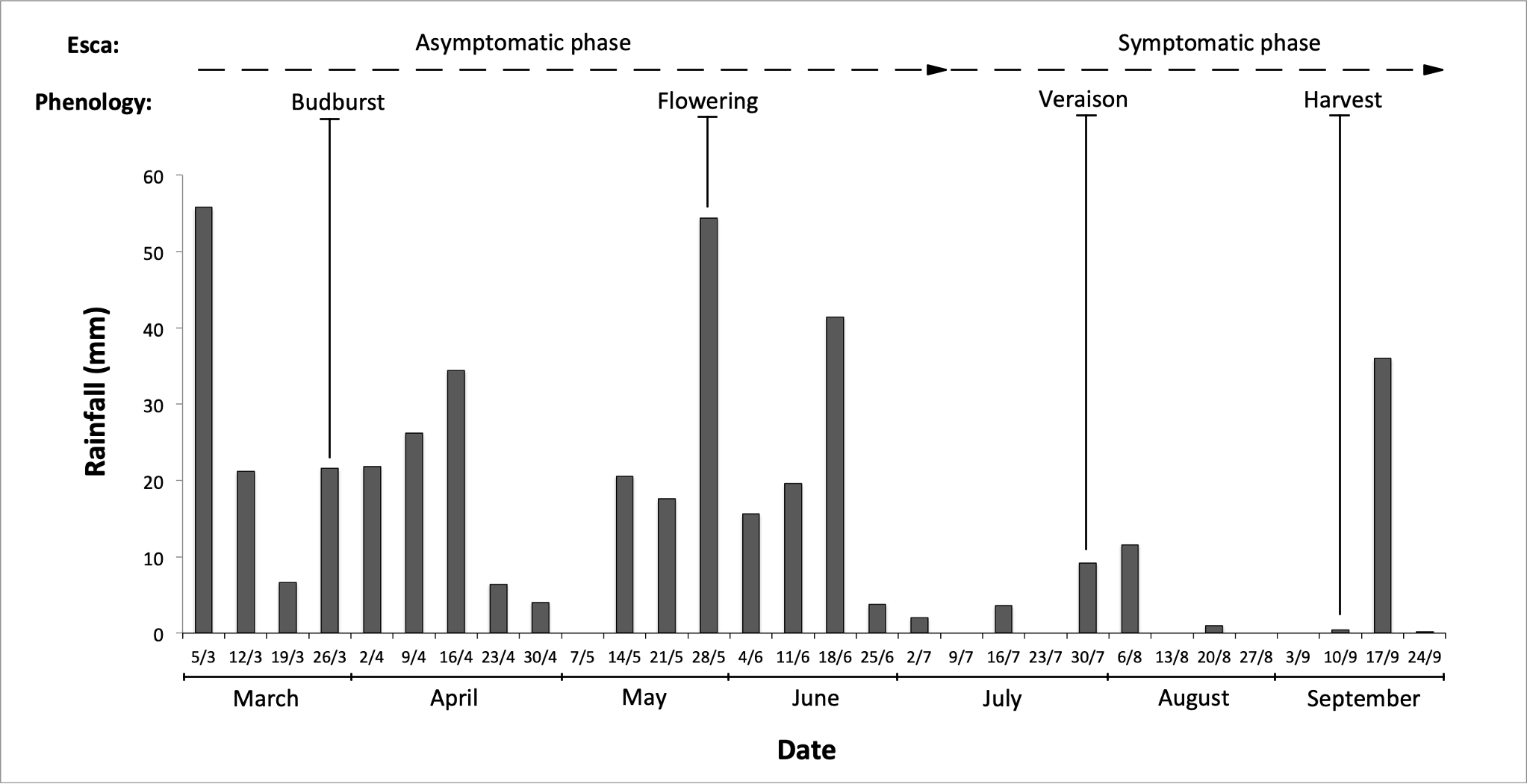

Supplement: S2 Fig — (TIF) [file pone.0222586.s002.tif]
